# A Study on the Effectiveness of College English Teaching Based on Content-Based Instruction Teaching Philosophy

**DOI:** 10.3389/fpsyg.2022.921654

**Published:** 2022-07-07

**Authors:** Lu Zhang, Qian Li, Wei Liu

**Affiliations:** School of English Language and Literature, Xi’an Fanyi University, Xi’an, China

**Keywords:** CBI teaching concept, college English teaching, teaching effectiveness, task-based teaching, education

## Abstract

With the development of world globalization, English plays an increasingly important role. English is the international language used in foreign communication, so English learning has become more and more important. Therefore, the innovation and research of College English teaching has become a hot spot in the field of education. Relevant research results are also constantly updated, but there are no ideas and methods that can adapt to the actual teaching. To improve students’ English level and cultivate excellent English talents for the development of the country, the CBI teaching concept aims to completely combine language learning with subject learning, so as to get rid of the artificial separation between language teaching and subject teaching in most educational backgrounds. This study constructs and implements the college English teaching model based on CBI teaching concept from an empirical perspective. In the teaching, it focuses on the theme content that students are interested in. Teachers take explaining the theme content as a means, use content teaching to drive language teaching, and realize the purpose of language teaching. The experimental class adopts CBI teaching concept, the blank class adopts ordinary teaching methods, and the control class adopts task-based teaching concept. Through one semester of teaching, the teaching effect of three classes is evaluated. Under the evaluation of teachers, the full score of CBI teaching effectiveness is 66. Under the evaluation of students, the full score of CBI teaching effectiveness is 64, which is the highest. This shows that the effectiveness of teaching under CBI is higher than task-based teaching and general teaching. The results showed that the average score of the pretest control class was the highest, the average score of the middle test experimental class was the highest, an increase of 4.7 points, and the average score of the post-test experimental class was the highest, an increase of 112.2 points, whereas the scores of the control group and the blank group were 108.7 and 104.3 points, respectively. This shows that the learning effect of CBI teaching concept is higher than task-based teaching and general teaching. Therefore, college English teaching based on CBI teaching concept has high effect, can improve students’ English knowledge and ability, and can be promoted in college English teaching.

## Introduction

The rapid development of China’s foreign trade and economy needs a large number of English majors. International exchanges and cooperation substantive listing need English majors to contact and exchange. College English teaching also needs to pay attention to cultivating students’ communication and cooperation skills. However, at present, students’ English level is uneven, and their reading ability and oral ability are relatively weak (Branzila, 2020). There are some problems in college English teaching at present. The traditional old teaching methods make students only learn grammar and words, which not only has a narrow range of knowledge but also ignores the cultivation of students’ reading ability and interest. The teaching concept of CBI advocates the combination of content learning and language learning (Huadan and Xin, 2020). This paper applies CBI teaching concept to college English teaching practice, which provides a new idea for college English teaching.

Content-based instruction (CBI) is known as content-based teaching. As a main teaching method, it has attracted the attention of many scholars and professors. Huang made a research on the application of CBI teaching method in English learning and found that CBI teaching method can help to improve English learners’ English learning ability (Huang, 2019). Y tries to solve this gap by applying the concepts of “curriculum genre” and “task structure,” and to observe and analyze the influence of L1 and L2 on the teaching effect of English teaching in Hong Kong middle schools (Lo and Lin, 2019). His research enriches the CBI teaching concept, so that we have a deeper understanding of it.

The application of new media technology in BE teaching is the practice of technological innovation. Zheng (2019) discusses several ways to promote BE teaching reform and innovation using new media technology, aiming to explore new ways to deepen BE teaching reform and improve BE teaching quality. Xie reports on the application of case-based teaching method in BE course. Through open-ended questionnaire survey and researcher’s participation, observation, and reflection, the author expounds the participants’ views and puts forward suggestions for the improvement in the teaching process (Xie, 2017). Their research provides a new way for BE teaching, but it still does not solve the problem of students’ enthusiasm in class.

Based on the CBI teaching concept, this paper studies the effectiveness of CBI teaching. The innovations of this study are as follows: (1) a comparative experiment was carried out. CBI teaching, task-based teaching, and general teaching were adopted in three classes with similar levels. (2) Questionnaire interviews with teachers and students were conducted to comment on the effectiveness of each class teaching mode. The data show that the effectiveness of CBI teaching behavior is higher than that of task-based teaching and general teaching. (3) CBI teaching concept is different from traditional teaching concept, which can effectively improve students’ linkage with other subjects and improve their learning ability.

The main framework of this paper is as follows: First, this paper describes the current situation of college English teaching and verifies the significance of this research through relevant research. Second, this paper introduces college English teaching and CBI teaching and analyzes the application of data mining technology in teaching evaluation according to the effect of college English teaching. After that, the college English teaching process based on CBI teaching is designed. Finally, comparing the designed CBI teaching with other traditional teaching methods, it is concluded that CBI teaching is effective in college English teaching.

## Content-Based Instruction Teaching Concept and College English Teaching

### Theoretical Basis of Content-Based Instruction Teaching Idea

Content-based instruction is a teaching idea rather than a specific teaching method, and it has no unified model. Some of the most common models are applied by more and more foreign language educators worldwide, including curriculum models, supplementary courses, subject-based, and regional research modules, language for special purposes (LSP), subject teaching, and interdisciplinary foreign language teaching (FLAC).

CBI has two different modes: content-based and language-based (Hong-yue, 2018; Jian, 2019). According to the different teaching objectives, CBI teaching mode can be divided into four types: theme, course, auxiliary, and special topic (Tajeddin et al., 2020). The curriculum model is highly professional and only suitable for higher education. The learning materials selected by the auxiliary mode are moderate in difficulty and can be combined with language teaching and professional knowledge teaching. The project model has a higher requirement for teachers, and students learn the language through the comprehensible input provided by professional courses. Based on these four models, “six T method” is derived (Zhen, 2018).

Krashen’s second language acquisition theory holds that to learn a second language well, it is necessary to create a second language acquisition environment similar to the learner’s mother tongue acquisition environment (Maguddayao and Tulauan, 2019; Rabie, 2021). The learning of this language material can be regarded as comprehensible language input, which does not need to follow the natural order of language acquisition.

The teaching practice of CBI also conforms to the learning theory of constructivism, which has a great continuity with cognitive learning theory and holds that learning is a process of meaning construction, in which learners form, enrich, and adjust their cognitive structure through the interaction of new and old knowledge and experience.

Content-based instruction teaching practice is also in line with the learning theory of constructivism. Learning is a process in which learners actively select, process, and process external information according to their own experience (Nosratinia and Fateh, 2017). Teachers no longer just ignore learners’ existing knowledge and experience to impart knowledge rigidly. Constructivism holds that knowledge is people’s interpretation and hypothesis of the objective world, and it will be constantly updated and deepened with the deepening of the cognitive process (Poehner and Rémi, 2018). In FLAC, it is very important to create language environment and design communicative activities.

### College English Teaching Mode

#### Cooperative Learning

Cooperative learning contains positive emotions, encourages language training, helps to master professional knowledge, and promotes the development of thinking and social skills (Pinying, 2018; Zhi-Qian, 2019). College English requires students to have good oral communication ability, so that they can better communicate with foreign English. College English requires students to be able to use English for business cooperation and communication, so it has a very high requirement for communicative competence. Students of college English should be more involved in practical activities to master relevant skills.

The traditional class teaching system makes students in a passive learning state, lack of initiative. In the teaching of College English, group learning has become one of the favorite learning methods for teachers and students (Radyuk and Pankova, 2017). But in the practice of group learning, there are always problems such as improper combination and poor order. Only by ensuring that the group has a common goal in learning and a perfect management system in class, can the effect of group learning be brought into full play. Cooperative learning with high structure can achieve these requirements and ensure good teaching effect.

#### Task-Based Teaching Method

Task-based approach transforms the basic concepts of language use into the practice of classroom teaching, and its task is to carry out targeted communication activities (Pang, 2019). Task-based approach is compatible with communicative framework and emphasizes that learners should carry out communication activities based on the understanding classroom input. This teaching method can let learners have the opportunity to use all kinds of things in real life to learn and use the language and improve their language ability.

Task-based approach embodies humanism and solves the contradiction between meaning and form of language in theory. Task-based approach emphasizes interaction and communication through the use of the target language and introduces the real context into learning (Sela and Luke, 2020). To provide learners with the opportunity to master the learning process, in the process of learning by enhancing the experience of learners to improve the enthusiasm of learning, task-based approach combines classroom language with extracurricular language to improve learners’ language use ability and thinking ability.

The teaching environment of task-based teaching method is more real, which can improve classroom efficiency and give play to learners’ subjective initiative. Students cannot only complete the task but also find their own interest in learning. Students need to use the existing knowledge and skills to solve the problems in the learning process, so the application ability and thinking ability will be developed. The range of activities is wide, involving rich and diverse information, which can broaden the scope of students’ knowledge.

#### Project Teaching Method

The task-based teaching method is the same as the action-oriented teaching method. But the purpose of project teaching method is to let students use the knowledge to solve problems in the process of completing the project (Yan, 2019). This needs to be formulated according to the actual work needs, and the original knowledge structure needs to be broken when arranging the course. Students can freely form a project team to understand and use the knowledge and skills they have learned in the process of practice and feel the joy of fun and solving difficulties.

The characteristics of project teaching method are reflected in that the scenario design of teaching content should be based on the logical requirements of practical work, and students should learn independently and comprehensively use knowledge and skills (Guzikova et al., 2018). It should be noted that the project used in project teaching method must be suitable for the actual situation of students, the project is operable, and the judgment of process and effect is open.

### Evaluation of Teaching Effectiveness

#### Teaching Effectiveness

The effectiveness of teaching can be defined from the input–output relationship of teaching, students’ development orientation, hierarchical deconstruction, and new curriculum concept (Ginaya et al., 2019).

The factors that affect the effectiveness of teaching can be summarized from two aspects: teaching theory and teaching practice. In terms of teaching theory, curriculum design and arrangement, teaching quality, content and environment, personal factors of teachers and students, teachers’ professional interest, concept, and sense of responsibility will affect the effectiveness of teaching (Nguyen et al., 2020). In terms of teaching time, due to the combination of teachers’ and students’ activities, their thinking ability and learning methods will affect teachers’ teaching efficiency and effect.

#### Evaluation Criteria of Teaching Effectiveness

The effectiveness of teaching includes the effectiveness of teachers’ teaching and the effectiveness of students’ learning (Domínguez Izquierdo, 2021). First of all, the teacher’s expression of knowledge in class should be clear and easy for students to understand. The effectiveness of blackboard writing requires that the content of blackboard writing is the key and difficult point of teaching, and the writing of blackboard writing is clear and neat. The effectiveness of multimedia teaching requires that the selected multimedia materials must be able to guide students to perceive the teaching content and cultivate their thinking ability. The validity of question-answering behavior requires that the difficulty of the question is appropriate and the students have equal opportunities to answer the question. The effectiveness of the creation of teaching situation requires the creation of an orderly, cooperative, and interactive classroom environment. The effectiveness of classroom homework management requires that the difficulty and quantity of homework should be appropriate, and timely feedback and comments should be made on homework.

From the perspective of three-dimensional objectives, the effectiveness of students’ learning is the effectiveness of knowledge and skills. Students should master effective knowledge and finish their homework. The second is the effective grasp of process and method. The proportion of students’ practice and learning engagement should be appropriate, and students’ knowledge should be timely feedback and correction. Finally, the effectiveness of students’ mastery of emotions, attitudes, and values, students should experience the joy of learning in the process of learning and obtain spiritual harvest.

#### Application of Data Mining in Teaching Evaluation

The evaluation results of teaching effect can be divided into several evaluation results and related analysis composed of similar objects, so that the relevant information can be obtained quickly and efficiently (Wadsorn, 2019). Euclidean distance, Mahalanobis distance, and absolute value distance are commonly used in cluster analysis. Their calculation is shown in Formulas 1, 2, and 3, respectively:


(1) $$d_{i\,j}={\left[{\left(a_{i}-a_{j}\right)}^{t}\,\left(a_{i}-a_{j}\right)\right]}^{\frac{1}{2}}$$

(2) $$d^{2}={\left(a_{i}-a_{j}\right)}^{T}\,S^{-1}\,\left(a_{i}-a_{j}\right)$$

(3) $$d_{i\,j}={\left|a_{i}-a_{j}\right|}^{T}$$

In the clustering of the shortest distance method, first, the distance between classes is the distance between the two nearest samples, as shown in Formula 4:


(4) $$D_{k\,l}=\min_{i\in E_{k},j\in E_{l}}d_{i\,j}$$

where _*D_kl_*_ is the smallest element, and _*E_k_, E_l_*_ are two categories, respectively. The recursion of the distance between the synthesized new class and any one is shown in Formula 5:


(5) $$D_{n\,j}=\min_{i\in E_{n},j\in E_{j}}d_{i\,j}=\min\left\{D_{n\,j},D_{l\,j}\right\}$$

In the clustering of the longest distance method, first, the distance between classes is the distance between the two farthest samples, as shown in Formula 6:


(6) $$D_{k\,l}=\max_{i\in E_{k},j\in E_{l}}d_{i\,j}$$

The distance recursion is shown in Formula 7:


(7) $$D_{n\,j}=\max\left\{D_{n\,j},D_{l\,j}\right\}$$

Association rule is also one of the widely used data mining methods. When the association rule is _*X⇒Y*_ and the data set is _*D*_, the calculation formulas of support, confidence, and expected confidence are as follows:


(8) support(X⇒Y)=|{F:X∪Y∈F,F∈D}|/|D|=sup⁡p⁢o⁢r⁢t⁢(X∪Y)

(9) confidence(X⇒Y)=support(X∪Y)/support(X)

(10) ex-confidence(X⇒Y)=support(Y)

## College English Teaching Experiment Based on Content-Based Instruction

### Subjects

The research object of this paper is college English teaching under the CBI teaching concept. The sample is sophomores majoring in college English in a university, because sophomores have certain business knowledge and English foundation. There are 87 sophomores in this major, and they are divided into three classes, 29 in the experimental class, 29 in the control class, and 29 in the blank class, and the questionnaire set up is shown in the Appendix of this paper.

### Experimental Methods and Tools

**FIGURE 1:**
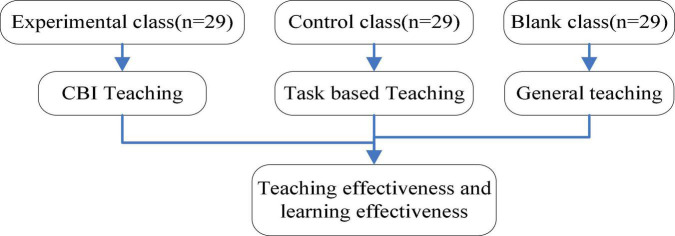
Research roadmap.

As shown in Figure 1, the experimental class adopts the CBI teaching concept to teach college English, the control class adopts the task-based teaching concept, and the blank class adopts the common teaching method for one semester. The teaching effectiveness of the three classes is evaluated, including the effectiveness of teaching narration, blackboard writing, multimedia teaching, question and answer, discussion, situation creation, homework, and other behaviors. The pretest, middle test, and post-test are used to test students’ college English knowledge and ability. For the experimental effect, this paper adopts the group scoring system, with the full score of each index being 10 points. The higher the score, the better the effect.

As shown in Figure 1, CBI teaching mode provides a new idea for college English classroom teaching reform because of its simple operation, relatively low requirements for students’ language level, and effective utilization of the advantages brought by multimedia and network technology. The teaching materials of the model are selected from various original themes and topics of the target language, such as the economy, politics, family, food, music, and so on, of a country. In CBI, these themes and topics play a central role. Learning grammar is no longer the center of teaching, and it revolves around and depends on topics. First of all, teachers combine topics with students’ prior knowledge, and teachers and students jointly identify and share content and curriculum objectives. According to learners’ different learning habits and traditions, teachers present the course contents in a variety of ways from the visual and auditory perspectives. Encourage students to use appropriate technical vocabulary to express the subject content and provide books, information texts, and other content-related resources and teach them how to use them. Teachers can realize the purpose of language teaching by preaching the theme content. CBI is conducive to stimulating students’ thinking about language forms, and it is conducive to students’ natural integration of listening, speaking, reading, and writing skills.

## Discussion on the Effectiveness of College English Teaching Based on Content-Based Instruction

### Effectiveness of Teaching Behavior

To evaluate the effectiveness of teaching behavior, including teaching narration, blackboard writing, multimedia teaching, question and answer, discussion, situation creation, classroom homework, and so on, let the teachers and students score the effectiveness of each behavior, respectively, with a full score of 10. The higher the score, the better the effectiveness. The effectiveness of teaching behavior among experimental class, control class, and blank class was compared.

As shown in Table 1, under the evaluation of teachers, teaching presentation, multimedia teaching, and question and answer scores were all higher than in the other two groups, the full score of teaching behavior effectiveness of the experimental class using CBI teaching is 66, the full score of the control class using task-based teaching is 62, and the full score of the blank class using ordinary teaching is only 55. This shows that the effectiveness of college English teaching under CBI is higher than task-based teaching and general teaching.

**TABLE 1:** Effectiveness evaluation results of teachers’ teaching behavior.

Teaching behavior	Experimental class	Control class	Blank class
Teaching narration	10	9	8
Blackboard writing	8	9	9
Multimedia teaching	10	8	8
Question and answer	10	9	8
Discussion	10	10	7
Situation creation	9	9	7
Classwork	9	8	8
Total	66	62	55

**FIGURE 2:**
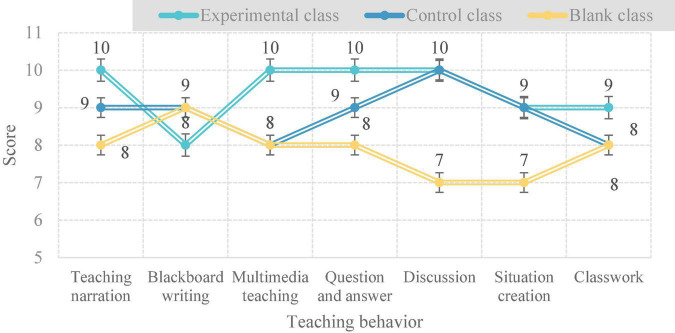
Teachers’ evaluation of the effectiveness of teaching behavior.

As shown in Figure 2, under the evaluation of teachers, the effectiveness of teaching presentation, multimedia teaching, question and answer, and classroom homework under the CBI teaching concept of the experimental class is the highest among the three teaching modes, with 10, 10, 10, and 9 points, respectively. The effectiveness of discussion and situation creation behavior is consistent with that of task-based teaching in the control class, which is higher than that of ordinary teaching. But the effectiveness of writing on the blackboard in CBI teaching is low, only 8 points.

**FIGURE 3:**
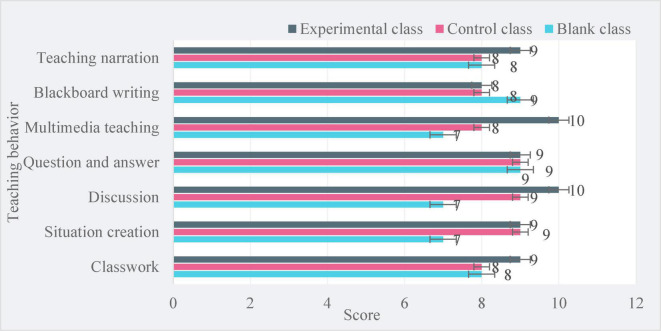
Students’ evaluation of the effectiveness of teaching behavior.

Then, the effectiveness of students’ teaching behavior was analyzed, 6 students were randomly selected, and 2 students from each class were selected to evaluate the effectiveness of teaching behavior. The results are as follows:

As shown in Figure 3, under the evaluation of students, students in the experimental group scored higher than those in the control group, especially in the multimedia teaching and discussion sessions. The full score of teaching behavior effectiveness of the experimental class using CBI teaching is 64 points, the full score of the control class using task-based teaching is 59 points, and the full score of the blank class using ordinary teaching is consistent with that under the evaluation of teachers, only 55 points. This shows that the effectiveness of college English teaching under CBI is higher than task-based teaching and general teaching.

### Learning Effectiveness

**FIGURE 4:**
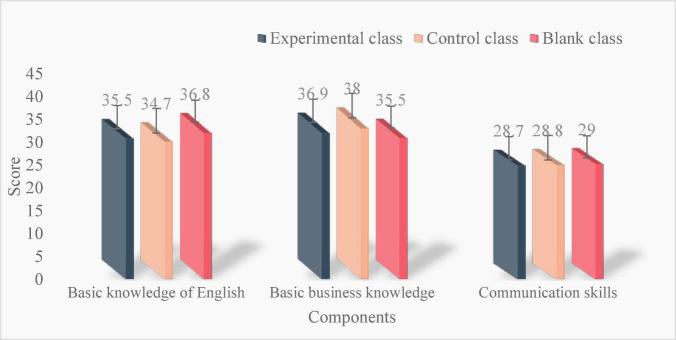
Pretest results.

**FIGURE 5:**
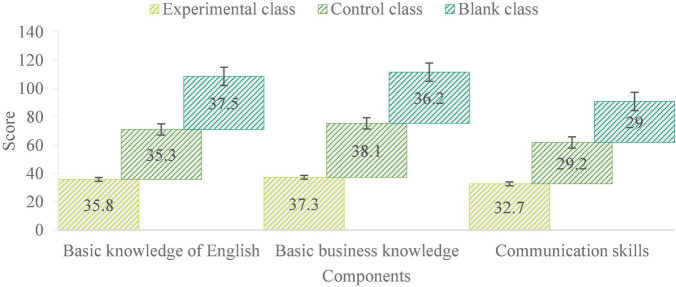
The results of intermediate test.

**FIGURE 6:**
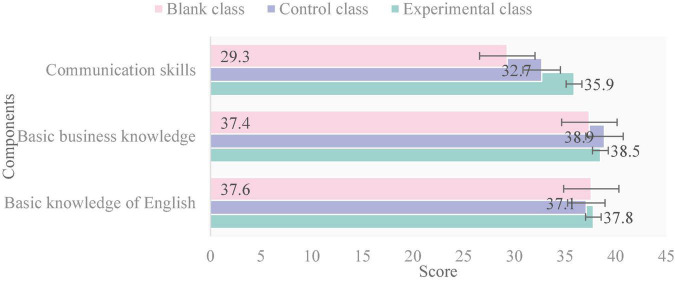
Post-test results.

The knowledge and ability of college English in the experimental class, the control class, and the blank class were tested before, during, and after. The level of college English knowledge and ability includes three parts: basic English knowledge, basic business knowledge, and communication ability. The full score of each part is 50 points, with a total of 150 points. The results of pretest are as follows:

As shown in Figure 4, the knowledge and ability of college English of the three classes are similar in the pretest, and the blank class has the highest score of basic English knowledge, which is 36.8. The control class had the highest score of 38 for basic English knowledge. The highest score of basic business knowledge was 38 in the control class. The highest score of communication ability was the blank class, which was 29. The results of intermediate test are as follows:

As shown in Figure 5, the knowledge and ability of college English of the three classes in the middle school test have been a little far away. The blank class got the highest score of basic English knowledge, which was 37.5. The highest score of basic business knowledge was 38.1 in the control class. The highest score of communication ability was 32.7 in the experimental class. This shows that the communication ability of the experimental class has been greatly improved. The results of post-test are as follows:

As shown in Figure 6, the knowledge and ability of college English of the three classes in the post-test have been greatly separated. The highest score of basic English knowledge was 37.8 in the experimental class. The highest score of basic business knowledge was 38.9 in the control class. The highest score of communication ability was 35.9 in the experimental class. This shows that the knowledge and ability of college English in the experimental class have been greatly improved after a semester of CBI instruction. The results of the three tests are as follows:

As shown in Table 2, the control class has the highest average score in the pretest, and the experimental class has the lowest, but the three classes have similar college English knowledge and ability. After half a semester of teaching, the experimental class had the highest average score in the middle test, increased by 4.7 points, the control class increased by 1.1 points, and the blank class increased by 1.4 points. After a semester of teaching, the average score of the experimental class increased to 112.2 points, whereas the control class and blank class only had 108.7 and 104.3 points. This shows that the learning effectiveness of CBI teaching concept is higher than that of task-based teaching and general teaching.

**TABLE 2:** Comparison of learning effectiveness.

Test type	Experimental class	Control class	Blank class
Pretest	101.1	101.5	101.3
Intermediate test	105.8	102.6	102.7
Post-test	112.2	108.7	104.3

There will be different operating methods in CBI teaching, such as immersion teaching method and theme teaching method. The application of content-based teaching method in English business classroom is to choose specific teaching methods according to students’ characteristics, business knowledge and language knowledge, and the business theme of each unit and then design teaching activities, so that students can actively participate in teaching activities.

## Discussion

Content-based instruction teaching provides new ideas for the reform of college English classroom teaching because of its simple operation, relatively low requirements for students’ language proficiency, and effective use of multimedia and network technology. Compared with previous studies on the improvement of traditional teaching, this paper compares the effects of traditional teaching and CBI teaching and draws the conclusion that CBI teaching is beneficial to college English learning.

This paper also studies the differences between basic knowledge of college English learning and basic business knowledge in teaching. It is found that using CBI teaching method can effectively arouse students’ learning enthusiasm and continuously improve students’ English level for a long time.

## Conclusion

The effectiveness of teaching can be defined from the relationship between input and output of teaching, students’ development orientation, hierarchical deconstruction, and new curriculum concept. The factors that affect the effectiveness of teaching can be summarized from two aspects: teaching theory and teaching practice. Therefore, the effectiveness of teaching includes the effectiveness of teachers’ English teaching and the effectiveness of students’ learning, so the improvement in students’ English level is closely related to the effectiveness of teachers’ teaching.

Based on the CBI teaching concept, this paper studies the effectiveness of college English teaching for sophomores majoring in college English in a university. The experimental class adopts CBI teaching concept, the control class adopts task-based teaching concept, and the blank class adopts common teaching method. One semester teaching was conducted to evaluate the teaching and learning effectiveness of the three classes. It can be seen from the above experiments that teachers’ teaching performance and students’ learning efficiency have been significantly improved under the CBI teaching concept. Therefore, the college English teaching method based on CBI is effective and can improve students’ college English knowledge and ability, which can be widely applied in college English teaching. However, there are some defects in the research of this paper. For example, in the experiment, only a few subjects are selected for the experiment, which makes the results not universal. Therefore, in the follow-up research, we will select a wider range and more sample data for analysis.

## Author Biography

LZ was born in Xi’an, Shaanxi, China, in 1985. She received the Master of Art in Applied Translation from the Open University of Hong Kong, China. She has been working in the education industry since she graduated. Her research interests include cloud English teaching approaches and Translation Strategy of English. E-mail: zhanglu@xafyxy.wecom.work. QL was born in Xi’an, Shaanxi, China, in 1982. She received her Master’s degree from Xi’an International Studies University, China. Now, she works in the School of English, Xi’an Fanyi University. Her research interests include cloud English and American literature and English teaching approaches. E-mail: paper_8583@126.com. WL was born in Luo Yang, He Nan, China, in 1984. She received her Bachelor’s degree from Xi’an Fanyi University, China. Now, she works in the School of English Language Learning, Xi’an Fanyi University. Her research interests include English language and literature and English teaching methodology. E-mail: 411527236@qq.com.

## Data Availability Statement

The original contributions presented in this study are included in the article/supplementary material, further inquiries can be directed to the corresponding author/s.

## Author Contributions

All authors listed have made a substantial, direct, and intellectual contribution to the work, and approved it for publication.

## Conflict of Interest

The authors declare that the research was conducted in the absence of any commercial or financial relationships that could be construed as a potential conflict of interest.

## Publisher’s Note

All claims expressed in this article are solely those of the authors and do not necessarily represent those of their affiliated organizations, or those of the publisher, the editors and the reviewers. Any product that may be evaluated in this article, or claim that may be made by its manufacturer, is not guaranteed or endorsed by the publisher.

## Appendix

**Appendix Table:**

Basic situation	Your grade?
	Your profession?
Attitudes towards CBI teaching	Is CBI teaching effective?
	Is CBI teaching fun?
	Would you like to continue teaching with CBI?
The effect of CBI teaching	Did your English score improve?
	Are you interested in learning English?
	Has spoken English improved?
